# Microbiological Characteristics and Clinical Outcomes of Periprosthetic Infections Following Oncologic Megaprosthetic Reconstruction: A Retrospective Cohort Study

**DOI:** 10.3390/pathogens15030306

**Published:** 2026-03-11

**Authors:** Vasileios Karampikas, Stavros Goumenos, Andreas G. Tsantes, Ioannis G. Trikoupis, Panayiotis Gavriil, Anastasios G. Roustemis, Alexandros Zikopoulos, Vasileios Petrakis, Dimitrios V. Papadopoulos, Petros Ioannou, Olga Savvidou, Vasileios Kontogeorgakos, Panayiotis J. Papagelopoulos

**Affiliations:** 1 First Department of Orthopaedics, School of Medicine, National and Kapodistrian University of Athens, 106 79 Athens, Greece; giannistrikoupis@gmail.com (I.G.T.); gavriilpan@gmail.com (P.G.); roustemis.anastasios@gmail.com (A.G.R.); alexzik12@gmail.com (A.Z.); olgasavvidou@gmail.com (O.S.); vaskonto@gmail.com (V.K.); pjporthopedic@gmail.com (P.J.P.); 2 Leeds Major Trauma Centre, Leeds Teaching Hospitals NHS Trust, Leeds LS1 3EX, UK; stgoumenos@gmail.com; 3 Laboratory of Haematology and Blood Bank Unit, University General Hospital “Attiko”, School of Medicine, National and Kapodistrian University of Athens, 106 79 Athens, Greece; 4 Microbiology Department, “Saint Savvas” Oncology Hospital, 115 22 Athens, Greece; 5 2nd University Department of Internal Medicine, Department of Infectious Diseases, University General Hospital Alexandroupolis, Democritus University Thrace, 691 00 Komotini, Greece; vasilispetrakis1994@gmail.com; 6 Second Department of Orthopaedic Surgery, School of Medicine, Konstantopouleio General Hospital, National and Kapodistrian University of Athens, 106 79 Athens, Greece; di_papadopoulos@yahoo.gr; 7 School of Medicine, University of Crete, 710 03 Heraklion, Greece

**Keywords:** periprosthetic joint infection, megaprostheses, biofilm-associated infection, polymicrobial infection, antimicrobial therapy, two stage revision, difficult-to-treat pathogens

## Abstract

Background: Periprosthetic joint infection (PJI) is a severe complication following megaprosthetic reconstruction in musculoskeletal oncology. This study aimed to evaluate outcomes of different surgical strategies for PJI in lower-limb megaprostheses and identify factors associated with treatment failure. Methods: We performed a retrospective cohort study of 29 consecutive patients treated for PJI after oncologic megaprosthetic reconstruction. Reinfection was analyzed using cumulative incidence functions (CIFs) with death treated as a competing event. Overall survival was assessed using Kaplan–Meier analysis. Univariable cause-specific Cox regression was performed for exploratory risk-factor analysis. Results: Among 29 patients, coagulase-negative staphylococci and *Staphylococcus aureus* were the most frequently isolated pathogens, and difficult-to-treat organisms were identified in a substantial proportion of cases. In patients managed with two-stage revision, the cumulative incidence of reinfection was 15% (95% CI: 0–30%) at 1 year and 30% (95% CI: 10–50%) at 2 and 5 years. In the DAIR cohort, the cumulative incidence of reinfection was 25% (95% CI: 0–62.5%) at 1 and 2 years and 37.5% (95% CI: 12.2–75%) at 3 years. Positive reimplantation cultures occurred in 38% of cases and were strongly associated with subsequent treatment failure. Polymicrobial infections were present in 27.5% of cases. Amputation was required in 17.2% of patients, often due to multidrug-resistant organisms or poor soft tissue. Conclusions: Two-stage revision remains the most effective strategy for PJI management in megaprostheses. DAIR may control acute infection but is suboptimal for definitive treatment. Individualized, multidisciplinary approaches are essential to improve outcomes in this high-risk population.

## 1. Introduction

Limb salvage surgery using modular endoprosthetic reconstruction has become the standard of care for patients with primary or metastatic bone tumors of the extremities offering substantial functional and psychological benefits over amputation [1,2]. Periprosthetic joint infection (PJI) is one of the most devastating and difficult-to-treat complications and might entail multiple subsequent revisions, prolonged hospitalisation, or even amputation [3,4,5,6]. The incidence of PJI in oncologic megaprostheses ranges widely from 8% to 20%, far exceeding that observed in conventional joint arthroplasty [4,7,8,9]. After revision procedures of infected cases, the incidence of PJI can be up to 60% [2,7]. Potential factors leading to infection include immunosuppression, prolonged operating time, adjuvant treatments, poor soft tissue coverage and the extensive metallic surface of tumor prostheses [1,10].

Surgical management of PJI of megaprostheses is challenging as it often requires multiple revision surgeries, prolonged intravenous therapy and long-term rehabilitation [11]. The most commonly employed surgical options include debridement, antibiotics, and implant retention (DAIR), and staged revision procedures, either one-stage or two-stage exchange [12,13]. While DAIR is less invasive and preserves the existing prosthesis, its effectiveness is often limited to early or acute infections and selected patient populations [14,15]. In contrast, two-stage revision is widely regarded as the gold standard for chronic infections, offering more reliable infection eradication but at the cost of greater morbidity and treatment duration [16,17,18]. Current literature provides limited detail regarding pathogen distribution, the prevalence of difficult-to-treat organisms, and the prognostic significance of microbiological findings at reimplantation in this population [7,19]. Most existing reports emphasize surgical technique rather than microbiological characterization and antimicrobial strategy. As a result, the influence of organism profile and reimplantation culture status on subsequent outcomes in limb-salvage patients remains incompletely defined. Addressing these gaps is essential to inform surgical timing, antimicrobial selection, and surveillance strategies in this uniquely vulnerable cohort.

Aims of our study were (1) to evaluate the efficacy of surgical treatments and antibiotic regimens for eradication of periprosthetic infection of tumor prostheses of the lower limb in oncological patients and (2) to investigate potential factors that may lead to treatment failure.

## 2. Materials and Methods

### 2.1. Study Design

Between 2011 and 2022, a total of 248 patients with musculoskeletal tumors underwent limb-salvage surgery with subsequent megaprosthetic reconstruction in the largest referral center for musculoskeletal tumors nationwide. After receiving ethical board approval (ΕΒD484/28-06-2023), data were collected on patient demographics, tumor diagnosis, surgical treatment, adjuvant therapies, clinical outcomes, complications, and microbiological findings. The cohort included 113 males and 135 females, with a mean age of 58.2 years (range, 12 to 87 years). Tumor diagnoses included osteosarcoma (67 patients), chondrosarcoma (52 patients), Ewing sarcoma (20 patients), metastatic bone disease (71 patients), malignant giant-cell tumor (17 patients), and other sarcomas (21 patients). The perioperative antibiotic regimen consisted of 600 mg of intravenous teicoplanin administered 30 min prior to incision. Postoperatively, patients received 600 mg of teicoplanin once daily together with 4 (+1.5) g of piperacillin/tazobactam every 8 h until postoperative day five. For shoulder procedures, teicoplanin in combination with clindamycin was administered for the same 5-day period. considering the high infection risk associated with extensive oncologic resections, large implants, and patient-related risk factors. This regimen was intended as extended perioperative infection prevention rather than treatment of PJI, considering the high infection risk associated with extensive oncologic resections, large implants, and patient-related risk factors [20].

All procedures were performed by two orthopedic surgeons (P.J.P. and V.A.K.) specialized in musculoskeletal oncology. The reconstruction of bone and joint defects after tumor excision was performed using the Modular Universal Tumor and Revision System (MUTARS^®^, Implantcast, Buxtehude, Germany).

**Figure 1:**
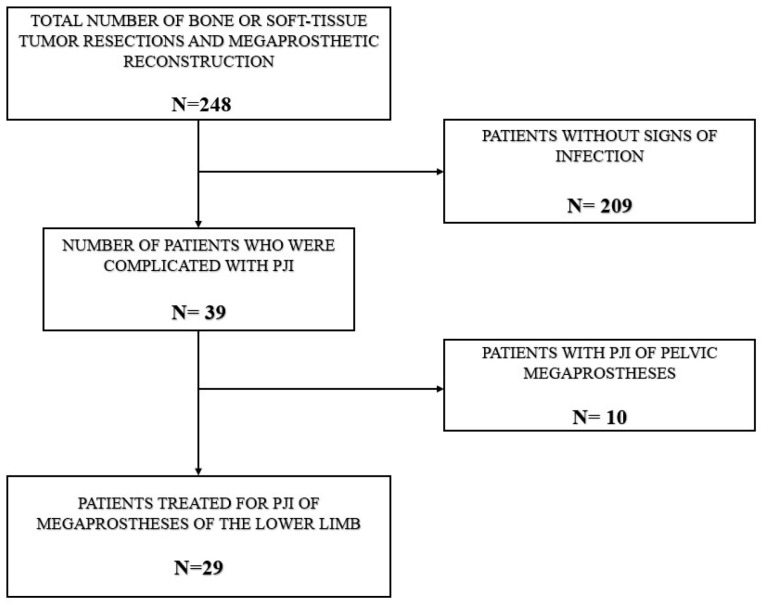
Flowchart illustrating patient selection and inclusion process. The final study cohort consisted of 29 patients treated for PJI of lower limb megaprostheses.

Inclusion criteria comprised adult and pediatric patients who underwent endoprosthetic reconstruction of the lower limb with modular oncological megaprostheses, including standard or lengthening implants, following bone or soft-tissue tumor resection and were diagnosed with first or recurrent periprosthetic infection after limb-salvage surgery. Exclusion criteria of our study were: (1) patients who underwent megaprosthetic reconstruction for non-oncologic conditions, (2) patients with pelvic tumors who were treated with internal hemipelvectomy and pelvic megaprostheses, due to inherent different nature of these surgeries and different microbiological flora implicated in these PJIs. This retrospective cohort study was conducted in accordance with institutional ethical standards and was reported in alignment with established guidelines for observational research. Out of 248 patients, 39 developed PJI, corresponding to an infection rate of 15.5%. Based on the exclusion criteria, ten patients who had undergone internal hemipelvectomy with pelvic reconstruction were excluded. Consequently, the final study cohort consisted of 29 patients (Figure 1). The mean time from index reconstruction to diagnosis of periprosthetic joint infection was 23.9 months (range, 0.5 to 120 months). The mean duration of follow-up following surgical treatment for infection eradication was 27.4 months (range, 2 to 96 months), with a minimum follow-up of 12 months. PJI was classified according to the pathogenesis-based framework described by Zimmerli et al. [21]. Infections were categorized as early (≤3 months after implantation) in 10 patients (34.4%), delayed (>3 to ≤24 months) in three patients (10.3%), while the remaining 16 cases (55.1%) were classified as late PJIs, presenting more than 24 months following the initial surgery. Classification was based solely on time from implantation to diagnosis to ensure mutually exclusive categories. Symptom duration was recorded descriptively but was not used to define infection stage.

### 2.2. Definitions of Infection and Treatment Failure

Infection was diagnosed based using the European Bone and Joint Infection Society (EBJIS) criteria for the definition of PJI by the same interdisciplinary, specialized team [22]. When these diagnostic methods were unavailable, infection was determined using clinical and laboratory findings or positive intraoperative cultures from revision surgery. Seventeen patients fulfilled the major criteria for a confirmed preoperative infection—twelve presented with a sinus tract communicating with the prosthesis, while five demonstrated markedly elevated leukocyte counts (≥3000 cells/µL) and polymorphonuclear cell percentages (≥80%). The remaining twelve patients were classified as having a likely infection. All of them showed positive results on preoperative aspiration cultures accompanied by elevated C-reactive protein (>10 mg/L (1 mg/dL) in blood workup, elevated leukocyte counts (>1500) and PMN percentages (>65%); six exhibited wound healing complications, three showed radiographic evidence of implant loosening, and four reported a recent history of fever. In this group, the diagnosis of infection was confirmed postoperatively, as all patients had either more than two positive intraoperative cultures or >50 CFU/mL of any organism detected in the sonication supernatant. Treatment failure was defined as infection recurrence or relapse involving the same or different microorganisms using the EBJIS criteria, sepsis-related death, or amputation due to infection.

### 2.3. Microbiological Methods

Our microbiological protocol involved collecting one fluid aspirate sample and at least five separate tissue samples from the deep surgical site, along with culturing the supernatant obtained after sonication of the infected implant. Fluid sample was collected through aspiration prior to skin incision and inoculated in aerobic and anaerobic blood culture bottles. In addition, one tissue specimen from the peri-implant region was submitted for histopathological analysis [23].

For pathogen identification, all postoperative tissue and fluid specimens were cultured on standard aerobic and anaerobic media, including blood agar, chocolate agar, and MacConkey agar. Sabouraud dextrose agar was used for fungal recovery, whereas Löwenstein–Jensen medium and Mycobacteria Growth Indicator Tubes were employed for mycobacterial isolation. Aerobic cultures were incubated at 35–37 °C for a minimum of 7 days, and anaerobic cultures were incubated for 14 days to detect slow-growing or fastidious organisms, with mycobacterial cultures extended up to 6 weeks. Anaerobic cultures were performed using pre-reduced media and incubated under strict anaerobic conditions. When available, sonication fluid cultures were processed according to established protocols and incubated under both aerobic and anaerobic conditions for the same duration. Initial pathogen identification relied on conventional biochemical assays such as catalase, coagulase, and oxidase tests. Additionally, prolonged incubation for *Cutibacterium* spp. was not implemented as shoulder prostheses are not included in our study group. Final species identification and antimicrobial susceptibility profiling were performed using the VITEK2 automated system, with interpretation according to European Committee on Antimicrobial Susceptibility Testing (EUCAST) guidelines (EUCAST versions 1.3–12.0, depending on the year of analysis). Histopathologic results from intraoperative deep tissue samples, based on the Krenn–Morawietz periprosthetic membrane classification (Types I–IV), were not correlated with microbiological findings. According to our sonication protocol, explanted prosthetic components were aseptically placed in sterile containers with 200 mL of Ringer’s solution. Samples were vortexed for 30 s, sonicated (40 kHz, 0.2–0.3 W/cm^2^) for 5 min, and vortexed again. Aliquots of 0.1 mL were plated onto aerobic and anaerobic media for quantitative culture. Growth of ≥50 CFU/mL in sonicate fluid was considered positive [22,24,25]. Mycobacterial cultures were included in the microbiological protocol to exclude rare prosthetic infections caused by non-tuberculous mycobacteria, which may occur in chronic infections and in immunocompromised oncologic patients.

Interpretation of culture results followed standardized PJI criteria. Low-virulence organisms were considered clinically relevant only when isolated from ≥2 concordant samples or when sonication fluid cultures yielded ≥50 CFU/mL, whereas a single positive culture with high-virulence pathogens was regarded as diagnostic of infection. These thresholds were consistently applied to differentiate true infection from contamination.

Infections were stratified according to the virulence profile of the isolated microorganisms into high-grade and low-grade infections [26]. High-virulence pathogens comprised *Staphylococcus aureus*, *Enterococcus* and *Streptococcus* species, aerobic Gram-negative pathogens, and *Candida* spp. In contrast, low-grade infections were attributed to low-virulence organisms such as coagulase-negative staphylococci (CNS). Difficult-to-treat (DTT) microorganisms were defined as those with proven resistance to effective biofilm-active antibiotic regimen, including rifampicin-resistant staphylococci, fluoroquinolone-resistant Gram-negative bacilli, enterococci and *Candida* spp. [22]. Rifampicin penetrates mature staphylococcal biofilms and is active against sessile, metabolically inactive bacteria, with no other antibiotic having comparable biofilm activity against staphylococci on implants. Similarly, fluoroquinolones are the only antibiotics with reliable biofilm activity against Gram-negative bacteria on implant surfaces. Concerning enterococci, no antibiotic agent has proven biofilm-active efficacy against these microbes and standard regimes are active against planktonic cells only [21,22,27,28]. On the other hand, *Candida* spp. is DTT because antifungal agents cannot reliably penetrate biofilm with implant removal being recommended when feasible [22,29]. Cases in which multiple distinct organisms were isolated from intraoperative cultures were categorized as polymicrobial infections. Detailed antibiograms were not systematically reported due to the retrospective design; however, antimicrobial therapy was consistently guided by susceptibility testing results.

All microbiological analyses were performed in an ISO 15189-accredited clinical laboratory, ensuring adherence to internationally recognized standards for analytical quality and diagnostic reliability [30].

### 2.4. Surgical Procedures

One-stage revision surgery was performed in a patient with late PJI who was deemed medically unfit for a two-stage procedure due to significant comorbidities. The decision was supported by the presence of adequate bone stock, satisfactory soft tissue coverage, and an isolated microorganism with known antibiotic sensitivity. This procedure involved removing all prosthetic components, including anchorage stems, followed by extensive debridement and irrigation before implanting a new prosthesis. Eight patients with early infections were managed with debridement, polyethylene exchange, and implant retention. One patient underwent amputation, while another required resection arthroplasty due to sepsis by multidrug-resistant infections. Two-stage revision surgery was performed in 18 patients, as it was the preferred approach for patients with delayed and late infections. In two-stage revisions, all patients received pathogen-specific antibiotic therapy tailored to the isolated pathogens, clinical status, radiological findings, and laboratory results.

Antimicrobial therapy was administered in accordance with established principles for the management of periprosthetic joint infection, consistent with IDSA 2013 guidelines and the EBJIS framework [22,28]. The most commonly used oral antibiotic regimen was rifampicin combined with doxycycline, cotrimoxazole, or ciprofloxacin/moxifloxacin for Gram-negative and polymicrobial infections. Rifampicin was not routinely administered during the period between stages and was typically initiated after definite reimplantation in cases of confirmed staphylococcal infection and stable soft-tissue condition. Rifampicin was used exclusively in combination with pathogen-directed antimicrobial agents and was never administered as monotherapy. Its use was strictly limited to combination regimens due to the rapid emergence of resistance when used alone. In our cohort, rifampicin was initiated only after adequate surgical source control and clinical stabilization, and only in cases where the causative organism was susceptible. Due to heterogeneity in timing and duration, rifampicin exposure was not analyzed as a separate variable between stages. Antibiotic regimens were tailored to pathogen identification and susceptibility, with individualized off-label therapies used in selected cases involving rifampicin-resistant or difficult-to-treat organisms at the discretion of the multidisciplinary team. Antifungal therapy consisted primarily of azoles, selected according to species identification and susceptibility testing.

All treatment decisions were made within a multidisciplinary team (MDT) framework, consisting of orthopedic oncologic surgeons, infectious diseases specialists, clinical microbiologists, and, when appropriate, plastic surgeons and oncologists. Antimicrobial strategies and surgical approaches were determined through consensus discussion.

### 2.5. Rationale for Dalbavancin Use

Dalbavancin was administered to selected eight patients with Gram-positive periprosthetic joint infections as adjunct therapy after two-stage revision in combination with pathogen-directed systemic antibiotics [31,32]. The standard dosing protocol consisted of 1500 mg administered on day 1 and repeated on day 8, providing therapeutic serum and bone concentrations for approximately 6–8 weeks. In one patient with polymicrobial infection an additional third dose on day 28 was used to extend antimicrobial coverage based on clinical evolution and multidisciplinary decision-making [33,34]. Indications for dalbavancin use included: (1) infections caused by difficult-to-treat Gram-positive pathogens (e.g., rifampicin-resistant staphylococci, *Enterococcus* spp.), (2) intolerance or contraindication to standard prolonged intravenous therapy, (3) need to facilitate outpatient antimicrobial management, or (4) limited oral treatment options following reimplantation. Adequacy of antimicrobial exposure was not directly measured, as no predefined pharmacokinetic targets were defined for dalbavancin dosing. Treatment decisions were based on clinical response and microbiological data. The use of dalbavancin in this context represents off-label application for periprosthetic joint infection with exploratory outcomes. All treatment decisions were made within a multidisciplinary team (MDT) framework, consisting of orthopedic oncologic surgeons, infectious diseases specialists and clinical microbiologists. Antimicrobial strategies and surgical approaches were determined through consensus discussion.

### 2.6. Data Analysis

Continuous variables, including patient age, BMI, C-reactive protein (CRP), and serum albumin levels, were expressed as mean values with standard deviation (SD). Categorical variables and group-specific rates were presented as percentages. Categorical variables were compared using the Fisher exact test, given the small sample size and the presence of expected cell counts below five. Continuous variables were compared using the Student *t* test or the Mann–Whitney U test, as appropriate based on data distribution. A *p*-value of <0.05 was considered statistically significant.

The primary endpoint was reinfection following surgical management of PJI. Because mortality is substantial in oncologic megaprosthesis cohorts and precludes the occurrence of reinfection, reinfection was analyzed using a competing-risk framework. Cumulative incidence functions (CIFs) were estimated using the Aalen–Johansen method, with death without prior reinfection treated as a competing event. Point estimates of cumulative incidence were reported at predefined time points (1, 2, 3, and 5 years, as applicable), and 95% confidence intervals were calculated using bootstrap resampling. Comparisons of cumulative incidence between treatment groups were performed using Gray’s test. To explore associations between clinical variables and reinfection, univariable cause-specific Cox proportional hazards regression models were constructed. Cause-specific modeling was selected to evaluate etiologic associations with reinfection, whereas CIF methodology was used to estimate absolute risk. Given the limited number of events, multivariable modeling was not performed to avoid overfitting, and all regression analyses are considered exploratory. Overall survival was analyzed using Kaplan–Meier methodology, with death treated as the event of interest. Survival probabilities were estimated at predefined time points. Confidence intervals were set at 95%. Data analysis was conducted using IBM SPSS Statistics, Version 29.0 (IBM Corp., Armonk, NY, USA).

## 3. Results

### 3.1. Patient Cohort and Overall Survival

This study included 29 patients who developed periprosthetic infections following limb-salvage surgery with megaprosthesis reconstruction of the lower limb. Among them, 13 underwent distal femur (DF) replacement, 6 had proximal tibia (PT) replacement, 7 received proximal femur (PF) reconstruction, 2 had total femur (TF) replacement, and 1 underwent distal tibia replacement (Table 1).

Overall patient survival in the cohort was 79.3% (95% CI: 59.6–90.1%) at one year, 62.1% (95% CI: 42.1–76.9%) at three years, and 58.4% (95% CI: 38.5–73.8%) at five years, according to Kaplan–Meier analysis. Among patients treated with DAIR, three of eight died within the first year. In the two-stage revision group, three out of four deaths occurred due to oncologic disease after two years of follow-up.

### 3.2. Reinfection and Cumulative Incidence Analysis

Reinfection rate of our cohort was 31% as 9 out of 29 patients experienced infection recurrence. Overall reinfection rate after two-stage revision and DAIR was 33.3% and 37.5% respectively. One case of one-stage revision, one case of amputation and one patient treated with resection arthroplasty were infection-free at latest follow-up after surgical management.

Surgical outcomes varied according to pathogen profile and antibiotic regimen. Among the 15 patients with staphylococcal periprosthetic joint infection, 8 patients (53.3%) received rifampicin-based combination therapy, typically when implant was retained or after reimplantation stage. Rifampicin use was not protocolized and was initiated only after adequate surgical source control and clinical stabilization at the discretion of the infectious diseases team. The exact interval between culture clearance and rifampicin initiation was not systematically recorded due to the retrospective study design; however, rifampicin was not administered during active bacteremia. In clinically stable patients, post-treatment surveillance cultures were not performed routinely. In all cases, rifampicin was started 5–7 days after surgery in combination with pathogen-directed intravenous antibiotics, administered for 2–6 weeks, followed by prolonged oral combination therapy. Oral regimens consisted of rifampicin (600 mg in the morning and 300 mg in the evening according to institutional clinical practice to improve tolerability during prolonged therapy) combined with an active oral agent selected according to antimicrobial for a median duration of 10 weeks. Partner agents included fluoroquinolones (*n* = 2), trimethoprim–sulfamethoxazole (*n* = 3), and minocycline (*n* = 3). The infection was controlled in 5 of the 8 patients treated with rifampicin-based regimens. No cases of emergent rifampicin resistance were observed during therapy. Three patients with rifampicin-resistant staphylococcal isolates received alternative combination therapies. Two of these patients—treated with minocycline plus trimethoprim–sulfamethoxazole and cloxacillin plus clindamycin, respectively—achieved infection control, whereas one patient treated with daptomycin combined with colistimethate experienced infection recurrence. Given the small sample size and heterogeneity of antibiotic regimens, no comparative analysis of rifampicin efficacy was performed. Most *Staphylococcus* isolates remained susceptible to rifampicin and glycopeptides, with methicillin resistance present in a subset; antimicrobial therapy was tailored to isolate-specific antibiograms. The microorganisms isolated in subsequent PJIs did not show a consistent resistance pattern related to the antimicrobial agents administered during the initial postoperative period.

Two out of five successful cases of polymicrobial infection and two-stage revision received combinations including trimethoprim–sulfamethoxazole (800/160 mg twice daily) with minocycline (100 mg twice daily) or trimethoprim–sulfamethoxazole with clindamycin (600 mg daily) for 10 weeks. In contrast, three patients who received regimens based primarily on rifampicin (600 mg AM/300 mg PM) in combination with azoles, quinolones, or β-lactams failed in most cases despite extended therapy.

Dalbavancin was administered in eight patients (27.5%) as adjunct therapy following two-stage revision. Indications for dalbavancin use were rifampicin resistance (*n* = 3), outpatient management facilitation (*n* = 3) and limited oral options (*n* = 2). Two patients were infected with methicillin-resistant *Staphylococcus epidermidis*, two patients with rifampicin-resistant CNS infection, one patient with Methicillin-resistant *Staphylococcus aureus* and three patients had polymicrobial infections. Concomitant antimicrobial agents included fluoroquinolones (*n* = 1), trimethoprim–sulfamethoxazole (*n* = 4) and other agents (*n* = 2). At final follow-up, infection control was achieved in four out of eight (50%) patients receiving dalbavancin. These included one patient with *Staphylococcus haemolyticus*, one with *Staphylococcus capitis*, one with MRSE and one with polymicrobial infection who had an additional third dose due to high-risk microbiological profile. No adverse events attributable to dalbavancin were observed.

Antifungal therapy duration was individualized, consisting of prolonged suppressive therapy for 14 months in one patient and finite courses of 12 weeks (*n* = 2) and 18 weeks (*n* = 1) in the remaining cases.

At final follow-up, 17 patients (58.6%) were alive and free of infection. Among these, 3 patients (10.3%) had undergone amputation, 1 patient (3.4%) was receiving chronic suppressive antibiotic therapy, and 13 patients (44.8%) retained a tumor megaprosthesis in situ. Sepsis at presentation was documented in 3 patients (10.3%). Among these patients, one death was attributed to infection-related causes, one was considered unrelated to infection, and one patient remained alive without evidence of infection at the latest follow-up. Overall, 3 patients (10.3%) died from causes related to periprosthetic joint infection, with two of them occurring in the setting of progressive prosthetic joint infection despite suppressive antibiotic therapy, and one due to infection recurrence and sepsis. The remaining deaths (*n* = 9; 31.0%) were related to oncologic disease progression or unrelated medical conditions.

### 3.3. Two-Stage Revision Outcomes

**Figure 2:**
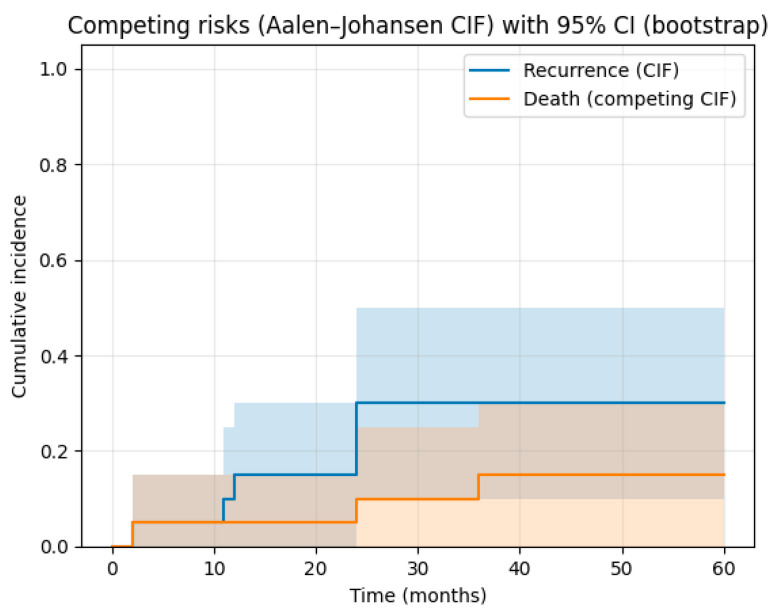
Cumulative incidence functions (Aalen–Johansen method) demonstrating the probability of recurrent infection after reimplantation, with death treated as a competing event. Shaded areas represent 95% confidence intervals estimated using bootstrap resampling. The lower curve represents the cumulative incidence of death without recurrent infection. Time zero was defined as the date of reimplantation.

**Figure 3:**
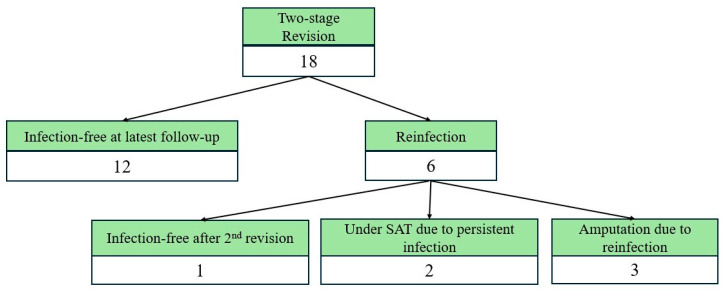
Clinical outcomes following two-stage revision for PJI in oncologic megaprosthesis. All values represent patients. Infection-free status includes patients with an implant in situ, including those receiving suppressive antibiotic therapy. Subsequent salvage procedures after reinfection are reflected in the final clinical outcome and are not shown as separate events. SAT = suppressive antibiotic therapy.

In the subgroup of patients treated with two-stage revision, at 1 year following reimplantation, the cumulative incidence of recurrent infection was 15% (95% CI: 0–30%). This increased to 30% (95% CI: 10–50%) at 2 years and remained stable at 30% (95% CI: 10–50%) at 5 years. (Figure 2). No further infection recurrences were observed beyond two years. Among those who experienced reinfection, two patients were treated with debridement and antibiotic suppression, while two underwent amputation. Two cases of failed two-stage revision were managed with a second two-stage revision, leading to one amputation, whereas the other patient remained infection-free (Figure 3). Following regression analysis evaluating risk factors for reinfection following two-stage revision for PJI, none of the examined variables reached statistical significance. A non-significant trend toward increased risk of reinfection was reported from increased age (HR = 1.08, 95% CI: 0.98–1.23, *p* = 0.09) and BMI (HR = 1.48, 95% CI: 0.92–3.59, *p* = 0.19). Chemotherapy was associated with a lower observed hazard of reinfection (HR = 0.05, 95% CI: 0.01–0.65). Given limited events and potential confounding by indication, this finding should be interpreted as exploratory. Variables such as polymicrobial infection, site of reconstruction, and spacer exchange strategies did not show significant associations.

### 3.4. DAIR Outcomes

**Figure 4:**
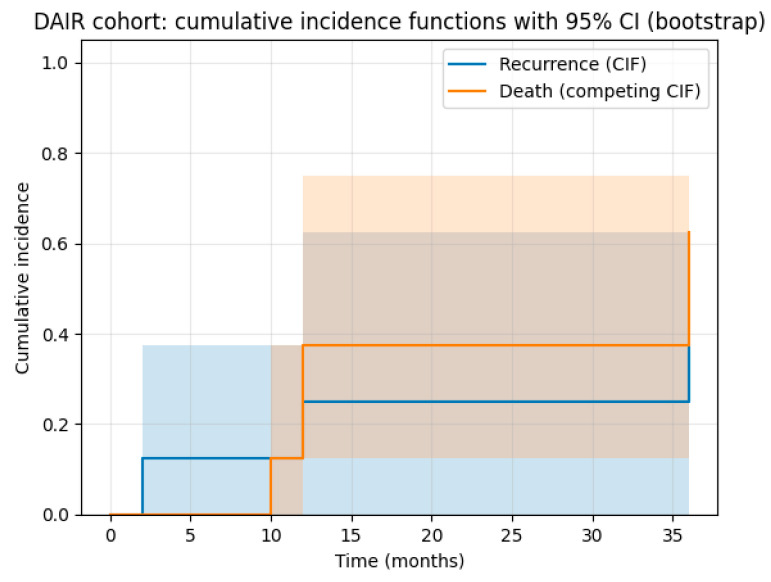
Cumulative incidence functions (Aalen–Johansen method) demonstrating recurrence of infection after DAIR, with death treated as a competing event. Shaded areas represent 95% confidence intervals estimated using bootstrap resampling. Time zero was defined as the date of index DAIR procedure.

Regarding patients managed with debridement, antibiotics, and implant retention (DAIR), the cumulative incidence of recurrence was 25% (95% CI: 0–62.5%) at 1 year and remained unchanged at 2 years. By 3 years, the cumulative incidence of recurrence increased to 37.5% (95% CI: 12.2–75%). (Figure 4). Two patients in this group ultimately required amputation due to insufficient bone stock and compromised soft tissue coverage, which rendered further revision surgery unfeasible. One patient with a proximal tibia replacement who experienced DAIR failure was later treated with a two-stage revision, remaining without signs of infection after eight years. Another patient treated with a one-stage revision remained free of infection at the five-year follow-up. The cumulative incidence of death without recurrence was 37.5% at 1 year and 50% by 3 years.

All patients treated with amputation or permanent explantation of prosthesis as the method of treatment of infection demonstrated no signs of infection recurrence until final follow-up.

### 3.5. Microbiological Profile and Infection Characteristics

**Figure 5:**
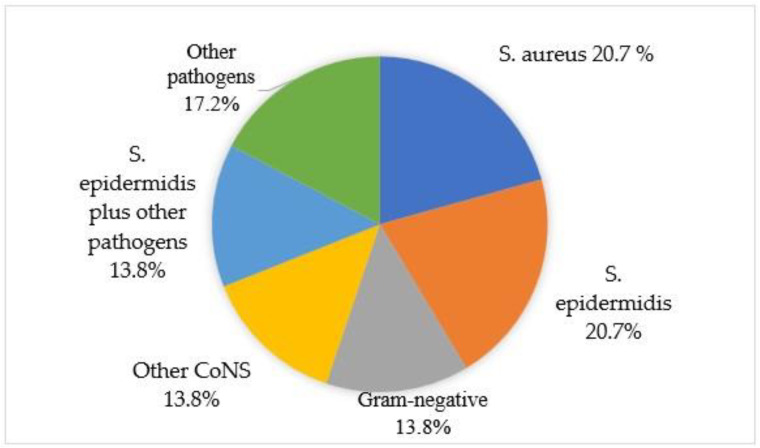
Causative pathogens for periprosthetic joint infections.

The most frequently isolated pathogens were coagulase-negative staphylococci (CNS) (*n* = 18; 62%), followed by *Staphylococcus aureus* (*n* = 8; 27.5%) and Gram-negative pathogens (*n* = 6; 20.6%). Regarding those infections from coagulase-negative staphylococci, the most common pathogen was methicillin-susceptible *Staphylococcus epidermidis* (*n* = 7, 24.1%), while methicillin-resistant *Staphylococcus epidermidis* was evident in three cases (10.3%). Among those eight patients with *Staphylococcus aureus* infections, methicillin-resistant *Staphylococcus aureus* was isolated in three (10.3%) patients, while methicillin-susceptible *Staphylococcus aureus* was isolated in five patients (17.2%). Additionally, the identified pathogens included enterococci in three patients (10.3%) and fungi in four patients (13.7%). The most common type of infection was a monomicrobial infection due to methicillin-susceptible *Staphylococcus aureus* (*n* = 5; 17.8%) followed by a monomicrobial infection due to methicillin-susceptible *Staphylococcus epidermidis* (*n* = 4; 14.2%) (Figure 5). Fungal infections included *Candida albicans* (*n* = 2), *Candida parapsilosis* (*n* = 1) and *Aspergillus* spp. (*n* = 1). The only isolated microorganism was fungus (*Candida albicans*) in one of these patients, while, in the other three cases, multiple pathogens such as *S. epidermidis* and *S. capitis* were isolated, with *Candida* spp. and *Aspergillus* spp. being one of them (Table 2).

In our cohort, 10 infections were classified as high-grade and 11 as low-grade. There was no significant difference in treatment success between groups, with infection control achieved in six of ten patients with high-grade infection and in eight of eleven patients with low-grade infection (*p* = 0.659). Patients with high-grade infection demonstrated higher mean C-reactive protein levels than those with low-grade infection, although the difference was not statistically significant (80.1 ± 26.7 mg/L versus 51.7 ± 38.8 mg/L; *p* = 0.077).

Six cases met the criteria for difficult-to-treat PJI, with three of them achieving at latest follow-up infection-free status. Notably, none of the cases had a culture-negative infection. Monomicrobial infections were evident in 21 (72.4%) patients, while polymicrobial infections (>1 pathogen) were evident in 8 patients (27.5%). At the latest follow-up, three of eight patients with polymicrobial infections remained infection-free, compared to 14 of 21 with monomicrobial infections (*p* = 0.832).

### 3.6. Impact of Positive Reimplantation Cultures

Positive intraoperative cultures at reimplantation were identified in 8 of 21 reimplantation procedures (38.1%), occurring in 8 patients who received prolonged, susceptibility-guided antimicrobial therapy. In three of these patients (3 of 8; 38%), only a single culture (1 of 5 samples) yielded a low-virulence organism, consistent with contamination according to EBJIS criteria. The remaining five patients (5 of 8; 62%) had ≥2 concordant positive cultures at reimplantation, indicating persistent infection. Four of these five patients (80%) subsequently experienced treatment failure and reinfection. In four cases, the microorganisms isolated at reimplantation were identical to those identified at the index infection. These included one patient with MSSE and *Candida* spp. at both index infection and reinfection; one patient with MSSE and MRSA at index infection who isolated MRSE at reimplantation and remained infection-free at final follow-up; one patient with *Staphylococcus capitis* and *Aspergillus* spp. at index infection who relapsed with the fungal pathogen and required long-term suppressive therapy; and one patient with MRSA, *Enterococcus faecalis*, and *Acinetobacter baumannii* at index infection who isolated MRSA at reimplantation and ultimately failed treatment. The remaining patient had a monomicrobial MSSA infection, with isolation of the identical organism at reimplantation and subsequent treatment failure. Patients with positive reimplantation cultures demonstrated a markedly higher incidence of reinfection compared with those with negative cultures.

### 3.7. Amputation and Limb Salvage

The overall amputation rate in our cohort was 17.2%, with five patients undergoing amputation for complete infection eradication. Two out of eight patients (25%) managed with DAIR were finally amputated due to infection recurrence, while only three out of 18 patients (16.6%) treated with two-stage revision underwent amputation (Chi-squared test, *p* = 0.62). In one case, amputation was the initial treatment approach.

## 4. Discussion

Managing periprosthetic joint infection of megaprostheses is a major challenge, as it is associated with high reinfection and amputation rates [12,35,36]. In a group of oncological patients who were treated in our hospital with tumor excision and reconstruction of the lower limb, an infection rate of 15.5% was reported. This finding is in accordance with previously reported results in similar patient populations, as infection rates range from 8% to 17% depending on tumor location, adjuvant therapy and prosthesis type [2,12,37,38,39]. Treatment strategies for managing PJI were guided by several factors, including the timing and classification of the infection, implant stability, the identified microorganism, the condition of the surrounding soft tissues, and the overall medical status of the patient. This study provides several contributions to the existing literature, including a detailed characterization of pathogen profiles in oncologic megaprosthetic PJI, the substantial prevalence of difficult-to-treat organisms, evidence that positive reimplantation cultures are strongly associated with subsequent treatment failure and outcome data derived from competing-risk methodology in a cohort with significant mortality risk. Together, these findings underscore the microbiological complexity and prognostic importance of reimplantation findings in this unique population.

DAIR with exchange of modular components was selected for acute infections when the implant remained stable, and when the causative pathogen was identified and susceptible to an orally administered antimicrobial agent. In this DAIR cohort, recurrence and mortality occurred at high and competing rates. By 3 years, 37.5% of patients experienced recurrent infection, while 62.5% died before recurrence. The relatively high competing mortality observed in this cohort suggests that implant- or infection-related durability must be interpreted within the broader context of patient frailty and systemic disease burden. In such populations, recurrence risk is substantial, but long-term implant survival is often limited by competing mortality rather than mechanical failure alone. Based on prior studies, success rates of DAIR in that group of patients range from 16% to 44.6% [14,15,17,36,40]. Similarly, Azamgarhi et al. found a 2-year infection-free survival 44.6% for DAIR with modular component exchange and only 24.7% without component exchange, highlighting the limited efficacy of DAIR, particularly when modular parts are retained [14]. In a recent study by McChesney et al., a 5-year reinfection rate of 16% for DAIR in patients with distal femoral or proximal tibial endoprostheses after tumor resection was reported [17]. Notably, this patient group was infected with highly virulent microorganisms and experienced elevated rates of amputation and sepsis-related mortality. However, one patient who experienced a recurrence three years after undergoing DAIR was successfully managed with a two-stage revision. In this high-risk group of oncological patients with megaprostheses, it seems that two-stage revision is the optimal and most widely supported treatment following failed DAIR, as it offers the best chance for infection eradication and limb salvage [17,41].

**Figure 6:**
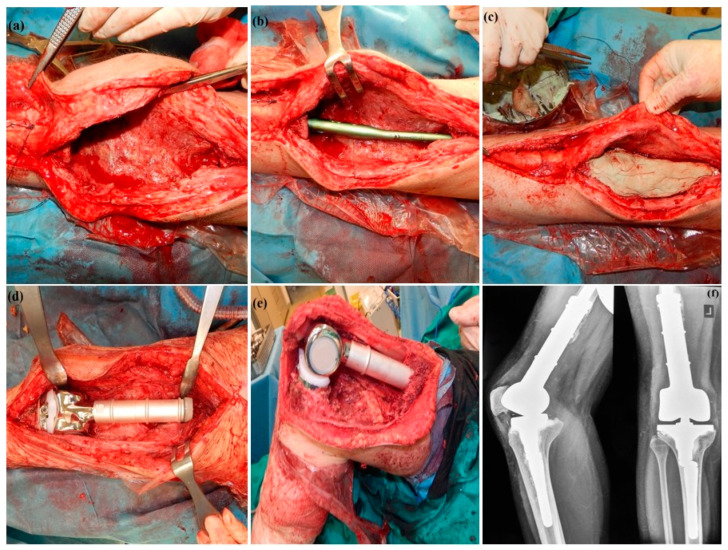
A 28-year-old female with a history of distal femoral replacement for osteosarcoma, performed 9 years prior, presented with a chronic periprosthetic joint infection (PJI). (**a**) Intraoperative view of the first stage demonstrating complete removal of the infected implant and extensive debridement of infected and devitalized soft tissues; (**b**,**c**) Fabrication and implantation of a static antibiotic-loaded cement spacer molded around a long tibial intramedullary nail to maintain limb length, provide mechanical stability, and ensure local antibiotic delivery during the interim period. (**d**) Intraoperative view of the second stage showing implantation of a silver-coated modular distal femoral replacement (MUTARS^®^, Implantcast GmbH, Buxtehude, Germany) following repeat irrigation and debridement; (**e**) Final reconstruction with restoration of limb length, alignment, and extensor mechanism continuity, allowing for functional knee flexion up to 90 degrees; (**f**) Postoperative anteroposterior and lateral radiographs demonstrating proper positioning and fixation of the silver-coated distal femoral megaprosthesis.

Two-stage revision for managing PJI in megaprostheses shows a wide range of short-term success rates, from 55% to 91%, and long-term infection eradication rates of approximately 60% to 74% [14,16,39,42,43]. These outcomes are notably lower than the 85–95% eradication rates typically reported for two-stage revision in conventional, non-oncologic total joint arthroplasty [44]. In this study, two-stage revision was employed as the principal treatment modality for periprosthetic joint infections occurring after one month of implantation, given the established formation of mature biofilm on prosthetic surfaces, which significantly limits the efficacy of implant retention strategies such as DAIR [45,46]. Furthermore, two-stage revision was also selected for late infections with less than 3 weeks of symptoms and failed DAIR procedures due to the compromised host status of oncological patients and the associated need for a more definitive and aggressive approach. This management strategy is supported by existing literature, which indicates that DAIR may be insufficient in such high-risk infections [17,45,47]. In this cohort of patients undergoing two-stage exchange for periprosthetic joint infection, the cumulative incidence of recurrent infection reached 30% at 5 years following reimplantation. Importantly, this estimate was derived using competing-risk methodology, accounting for death as a competing event. Conventional Kaplan–Meier methods would overestimate recurrence risk in this high-mortality oncologic cohort. This approach provides a clinically realistic measure of recurrence probability, particularly in populations with non-negligible mortality, closely mirroring outcomes reported in comparable oncologic populations. From a clinical perspective, these findings indicate that approximately one-third of patients undergoing two-stage exchange will experience recurrent infection within 5 years, while a smaller proportion will die prior to recurrence. A recent multicenter EMSOS study, including 186 patients treated with two-stage revision for infected megaprostheses, reported a reinfection rate of 39.1% and 50% at three and five years respectively [48]. In a retrospective study by Sigmund et al., reinfection rates were noted at 28% and 48% at two and five years, respectively, highlighting the risk of late recurrence [49]. Long-term follow-up data from McChesney et al. demonstrated a 75% infection-free survival at five years, while Azamgarhi et al. reported a 61% infection-free survival at four years in patients undergoing two-stage revision [14,17]. Additionally, it is important to emphasize that the objective of the two-stage revision approach was not only to eradicate biofilm from the prosthetic components and surrounding bone but also to address biofilm present within the soft tissues. Complete and meticulous soft-tissue debridement was therefore considered critical to achieving successful infection control. In cases involving resistant infections, an intermediate debridement and spacer exchange was performed between stages to further optimize the efficacy of the staged revision and enhance the likelihood of infection eradication (Figure 6).

All tumor prostheses implanted in our cohort were silver-coated. The use of silver-coated implants in two-stage revision for infected megaprostheses has been associated with slightly improved outcomes, including reduced reinfection and amputation rates. Although silver coatings are primarily intended for infection prevention, their application may also contribute positively to the management of established infections, offering potential therapeutic benefit in improving outcomes within this high-risk patient population [37,41]. However, other studies note that the use of silver-coated prosthesis during revision was not correlated with improved outcomes [14,49].

Primary amputation was performed as the initial treatment in one case of megaprosthesis infection due to the presenting septic condition and poor soft tissue coverage. Additionally, five patients required amputation following failed initial interventions with either DAIR or two-stage revision. These cases involved high-grade infections caused by antibiotic-resistant organisms, extensive osteolysis with prosthetic loosening, and inadequate soft tissue coverage, resulting in an overall amputation rate of 17.2% in our cohort. Comparable amputation rates of 21% and 24.4% following failed surgical management of PJI in megaprostheses have been reported by Mavrogenis et al. and Morii et al. respectively [45,50]. Although the differences were not statistically significant, our findings showed more favorable amputation rate for the two-stage revision group compared to the DAIR group (16.6% vs. 25%, *p* = 0.62). This trend is consistent with existing literature, which also reports higher rates of amputation in patients initially managed with DAIR [17,41].

The microbiological profile in our cohort, dominated by coagulase-negative Staphylococci and Staphylococcus aureus, is consistent with published literature [3,13,40]. Acute and early post-operative infections of megaprostheses are typically associated with more virulent pathogens, whereas late-onset prosthetic joint infections are generally caused by low-grade organisms. Staphylococcal species are the most frequently isolated pathogens in PJI, responsible for approximately 50% of cases, followed by streptococci, enterococci, *Enterobacteriaceae*, *Pseudomonas aeruginosa*, and various anaerobic bacteria [51]. Notably, *Enterococcus* species were linked to higher recurrence rates in a study, with the authors suggesting that implant removal is necessary for the eradication of infection [14]. The recent consensus statement from the European Bone and Joint Infection Society (EBJIS) on the management of acute periprosthetic joint infection underscored the significantly higher failure rates of DAIR procedures in cases caused by *Staphylococcus aureus* [44,52]. Based on this association, authors recommended preoperative microbiological identification whenever feasible, and suggested more aggressive surgical interventions including implant exchange in the presence of *S. aureus*, particularly in cases of late acute infections [44]. In our cohort, polymicrobial infections exhibited a non-significant trend toward increased reinfection rates relative to monomicrobial infections. Consistent with existing literature, infections caused by pathogens lacking susceptibility to biofilm-active antimicrobial agents, as well as polymicrobial infections, are associated with elevated reinfection risk [48]. The absence of culture-negative infections in this cohort is notable given the sample size. This finding likely reflects a high microbiological diagnostic yield rather than an atypical infection profile. At our institution, antibiotics are withheld preoperatively whenever feasible, and a standardized protocol is employed that includes systematic collection of multiple tissue samples, sonication of explanted components, and prolonged culture incubation. These measures may explain the lack of culture-negative cases observed in this series.

Molecular diagnostic techniques such as polymerase chain reaction (PCR) were not routinely employed in our cohort. Although PCR may increase diagnostic sensitivity in culture-negative or equivocal cases, it carries limitations including risk of contamination, detection of non-viable bacterial DNA, and limited standardization across laboratories [22,53]. These factors may complicate interpretation in biofilm-associated infections such as periprosthetic joint infection.

Positive reimplantation cultures were strongly associated with subsequent treatment failure in our cohort. This finding suggests that microbiological persistence at the time of reimplantation may represent incomplete infection eradication despite interval therapy. In the context of oncologic megaprostheses this may have prognostic relevance. Positive reimplantation cultures may warrant heightened surveillance and consideration of extended antimicrobial strategies. Although limited by small numbers, our findings are consistent with prior reports identifying positive reimplantation cultures as a risk factor for reinfection [54].

In PJIs involving oncologic megaprostheses, the absence of standardized antimicrobial treatment protocols reflects the biological and surgical complexity of these infections. High rates of multidrug-resistant organisms, extensive soft-tissue compromise, and large implant surfaces necessitate individualized, susceptibility-guided antimicrobial strategies integrated with surgical decision-making [28,37,55,56]. Due to small sample size and heterogeneity, antimicrobial regimens were considered descriptive variables rather than predictors of outcome.

This study has several limitations that should be considered when interpreting the findings. First, the retrospective design inherently introduces risks of selection bias and limits the ability to control for confounding variables, such as variations in host immune status, extent of soft tissue damage, and prior surgical history. The relatively small sample size of patients, especially those treated with DAIR, reduces the statistical power, particularly in subgroup analyses such as risk factor modelling and outcome comparisons. The heterogeneity of the patient population—with varying tumor types, reconstruction sites, and antibiotic regimens—further complicates the generalizability of the results. The modest sample size and limited number of events restrict statistical power and preclude reliable multivariable modeling. Reported associations should therefore be interpreted as hypothesis-generating rather than causal. While all surgeries were conducted by a specialized orthopaedic oncology team, institutional practices regarding timing of reimplantation and antibiotic regimens may differ from other centers. Lastly, the follow-up period, although sufficient for mid-term (2 to 5 years postoperatively) outcome evaluation, may underestimate late reinfections which are not uncommon in megaprosthetic reconstructions. These limitations underscore the need for multicentre, prospective studies to validate predictive factors and refine standardized protocols for the management of PJI in tumor endoprostheses.

## 5. Conclusions

Conclusively, the findings of this study highlight the considerable challenges associated with the management of periprosthetic joint infections following limb-salvage surgery with megaprosthetic reconstruction. Two-stage revision emerged as the most effective surgical strategy in achieving infection control and limb preservation, particularly in the setting of chronic infections, consistent with outcomes reported in comparable oncologic cohorts. In contrast, DAIR demonstrated limited effectiveness, as evidenced by elevated reinfection and amputation rates. While it may offer temporary control of the septic condition, it does not appear to be a reliable definitive treatment option for acute PJIs. The overall reinfection rate of 31% highlights the persistent risk of treatment failure, particularly in polymicrobial infections and those involving pathogens resistant to biofilm-active antibiotics. These findings emphasize the need for individualized, multidisciplinary treatment approaches and further highlight the importance of early diagnosis, pathogen characterization, and optimization of host and local factors.

## Figures and Tables

**Table 1:** Demographics of 29 patients with infected megaprostheses.

	*n* (%)
**Age (range)**	51.8 (11–81)
**Gender**	17 M–12 F
**Body mass index (BMI)**	26.12 kg/m^2^
**Duration of surgery (mean)**	4.7 h
**Inflammation markers at diagnosis (mean)** C-reactive protein White blood cell count	72.3 mg/L 8.5 × 10^3^/μL
**Tumor diagnosis** Osteosarcoma Chondrosarcoma Ewing sarcoma Metastatic bone disease Other	13 (44.8) 4 (13.7) 1 (3.4) 6 (20.6) 5 (17.2)
**Adjuvant therapies** Chemotherapy Radiotherapy	20 (69) 11 (37.9)
**Type of prosthesis** Proximal femur replacement (PF) Distal femur replacement (DF) Total femur replacement (TF) Proximal tibia replacement (PT) Distal tibia replacement (DT)	7 (24.1) 13 (44.8) 2 (6.8) 6 (20.6) 1 (3.4)
**Soft-tissue coverage** Good Medium Poor	9 (31) 11 (37.9) 9 (31)
**Type of fixation** Cementless Cemented	14 (48.2) 15 (51.7)
**Need for flap**	7 (24.1)

**Table 2:** Microorganisms isolated in patients with periprosthetic joint infection following megaprosthesis reconstruction.

Pathogens	Patients (*n* = 29)	Infection-Free at Latest Follow-Up (*n* = 20)	Reinfection (*n* = 9)
**Methicillin-resistant** ***Staphylococcus epidermidis*** **(MRSE)**	2 (6.8%)	1 (5.0%)	1 (11.1%)
**Methicillin-sensitive** ***Staphylococcus epidermidis*** **(MSSE)**	4 (13.7%)	3 (15.0%)	1 (11.1%)
**Methicillin-resistant** ***Staphylococcus aureus*** **(MRSA)**	1 (3.4%)	1 (5.0%)	0 (0.0%)
**Methicillin-sensitive** ***Staphylococcus aureus*** **(MSSA)**	5 (17.2%)	4 (20.0%)	1 (11.1%)
**Other isolated coagulase-negative** ***staphylococci*** **(CNS)** *Staphylococcus hominis* *Staphylococcus capitis* *Staphylococcus cohnii* *Staphylococcus haemolyticus*	1 (3.4%) 1 (3.4%) 1 (3.4%) 1 (3.4%)	0 (0.0%) 1 (5.0%) 1 (5.0%) 1 (5.0%)	1 (11.1%) 0 (0.0%) 0 (0.0%) 0 (0.0%)
**Gram-negative pathogens** *Enterobacter clocae* *Achromobacter* spp. *Klebsiella pneumoniae*	1 (3.4%) 1 (3.4%) 1 (3.4%)	1 (5.0%) 0 (0.0%) 0 * (0.0%)	0 (0.0%) 1 (11.1%) 0 (0.0%)
**Methicillin-sensitive** ***Staphylococcus epidermidis*** +*Candida parapsilosis* +*Staphylococcus warneri* +Methicillin-resistant* Staphylococcus aureus*	1 (3.4%) 1 (3.4%) 1 (3.4%)	0 (0.0%) 1 (5.0%) 1 (5.0%)	1 (11.1%) 0 (0.0%) 0 (0.0%)
**Methicillin-resistant** ***Staphylococcus epidermidis*** **+** ***Staphylococcus warneri + Enterococcus faecalis***	1 (3.4%)	0 * (0.0%)	0 (0.0%)
***Candida albicans***	1 (3.4%)	0 * (0.0%)	0 (0.0%)
***Streptococcus oralis***	1 (3.4%)	1 (5.0%)	0 (0.0%)
***Staphylococcus capitis*** **+** ***Candida albicans***	1 (3.4%)	0 (0.0%)	1 (11.1%)
***Staphylococcus capitis*** **+** ***Aspergillus*** **spp.**	1 (3.4%)	0 (0.0%)	1 (11.1%)
***Acinetobacter baumannii + Proteus mirabilis***	1 (3.4%)	1 (5.0%)	0 (0.0%)
**Methicillin-resistant** ***Staphylococcus aureus** + * ***Enterococcus faecalis + Acinetobacter baumannii***	1 (3.4%)	0 (0.0%)	1 (11.1%)

Values are presented as number of patients (%). Asterisks (*) indicate patients who were on chronic suppressive antibiotic therapy.

## Data Availability

The original contributions presented in this study are included in the article. Further inquiries can be directed to the corresponding authors.

## Abbreviations

The following abbreviations are used in this manuscript:

BMI	Body mass index
CRP	C-reactive protein
CI	Confidence interval
DAIR	Debridement, antibiotics, and implant retention
DF	Distal femur
DT	Distal tibia
EBJIS	European Bone and Joint Infection Society
EPR	Endoprosthetic reconstruction
PJI	Periprosthetic joint infection
PF	Proximal femur
PT	Proximal tibia
SD	Standard deviation
TF	Total femur
